# Tumor-Infiltrating Lymphocytes in Colorectal Cancer: The Fundamental Indication and Application on Immunotherapy

**DOI:** 10.3389/fimmu.2021.808964

**Published:** 2022-01-14

**Authors:** Ziyi Bai, Yao Zhou, Zifan Ye, Jialong Xiong, Hongying Lan, Feng Wang

**Affiliations:** ^1^ Key Laboratory of Molecular Medicine and Biotherapy, School of Life Science, Beijing Institute of Technology, Beijing, China; ^2^ College of Bioinformatics Science and Technology, Harbin Medical University, Harbin, China

**Keywords:** tumor-infiltrating lymphocytes, tertiary lymphoid structures, microsatellite instability, immunotherapy, colorectal cancer

## Abstract

The clinical success of immunotherapy has revolutionized the treatment of cancer patients, bringing renewed attention to tumor-infiltrating lymphocytes (TILs) of various cancer types. Immune checkpoint blockade is effective in patients with mismatched repair defects and high microsatellite instability (dMMR-MSI-H) in metastatic colorectal cancer (CRC), leading the FDA to accelerate the approval of two programmed cell death 1 (PD-1) blocking antibodies, pembrolizumab and nivolumab, for treatment of dMMR-MSI-H cancers. In contrast, patients with proficient mismatch repair and low levels of microsatellite stability or microsatellite instability (pMMR-MSI-L/MSS) typically have low tumor-infiltrating lymphocytes and have shown unsatisfied responses to the immune checkpoint inhibitor. Different TILs environments reflect different responses to immunotherapy, highlighting the complexity of the underlying tumor-immune interaction. Profiling of TILs fundamental Indication would shed light on the mechanisms of cancer-immune evasion, thus providing opportunities for the development of novel therapeutic strategies. In this review, we summarize phenotypic diversities of TILs and their connections with prognosis in CRC and provide insights into the subsets-specific nature of TILs with different MSI status. We also discuss current clinical immunotherapy approaches based on TILs as well as promising directions for future expansion, and highlight existing clinical data supporting its use.

## Introduction

Colorectal cancer (CRC) is a clinically common malignant tumor of the digestive system. According to Global Cancer Statistics of 2020, there are approximately 1.9 million newly diagnosed CRC patients and 935,000 CRC-related deaths, accounting for 10% of cancer cases and 9.4% of cancer-related deaths worldwide (1). With deeper understanding of pathophysiology in colorectal cancer, the optimization of screening and the application of various treatments have effectively improved the 5-year survival rate (2–5). However, nearly 40% of patients with CRC end up relapsing, with recurrent or advanced metastasis. As a result, extensive researches are now being conducted to overcome the barriers to relapse and resistance, and to explore more effective targets.

In the past decade immunotherapy has achieved impressive success in eradicating malignant cells by harnessing the inherent mechanisms of the host immune system, transforming the therapeutic landscape for a variety of solid and hematological malignancies (6, 7). Among cancer immunotherapy strategies, immune checkpoint blockade has shown significant benefits. It is the most thoroughly studied class of immunotherapy to date, increasing the overall survival (OS) rates of patients with advanced melanoma, non-small-cell lung cancer (NSCLC), urothelial cancer (8–10). Immune checkpoint therapy rejuvenates T cells and allows the adaptive immune system to block immune escape caused by cascade activation of tumor-specific immune checkpoints, such as those controlled by programmed cell death protein (PD-1), programmed death-ligand 1 (PD-L1) or cytotoxic T lymphocyte-associated protein 4 (CTLA-4) (11, 12). In the treatment of CRC, the PD-1 inhibitors pembrolizumab and nivolumab, which have been approved by the Food and Drug Administration (FDA), led a to durable response in patients with metastatic CRC that is mismatch-repair-deficient (dMMR) and microsatellite instability-high (MSI-H) (dMMR-MSI-H). Another inhibitor, ipilimumab, a fully-humanized monoclonal antibody that blocks CTLA-4, has also been approved by the FDA for combination with nivolumab in patients with dMMR-MSI-H CRC who have previously received chemotherapy.

According to The Cancer Genome Atlas Project’s CRC study based on the array and sequencing technologies, CRC can be classified into two main types: (1) ~ 16% hypermutated (>12 mutations per 10^6^ DNA bases) cancers with dMMR-MSI-H signature and (2) ~ 84% percent non-hypermutated (<8.24 mutations per 10^6^ DNA bases) with mismatch-repair-proficient (pMMR) and have low levels of microsatellite instability (MSI-L) or microsatellite stable (MSS) (pMMR-MSI-L/MSS) signature (13). Patients with pMMR-MSI-L/MSS have a worse prognosis than dMMR-MSI-H (14), and show unsatisfied responses to immune checkpoint inhibitors (ICIs) (15). In general, pMMR-MSI-L/MSS have low TMB, are often poorly infiltrated by TILs. Accumulating evidence has shown that tumor mutation burden and tumor-infiltrating-lymphocytes (TILs) correlate with ICIs response (16–18). Therefore, it is important to understand the relationship between genetic heterogeneity and the molecular level of TILs in CRC. In this review, we discuss the accumulating evidence about the fundamental feature of TILs and their prognostic value in the tumor micro-environment of CRC. We also review the clinical development of immune checkpoint inhibition in CRC and discuss the emerging clinical therapies for targeting TILs. Collectively, this work clarifies some aspects of TILs subsets discrepancy, which provides a scientific basis for a better understanding of the excessive interactions between immune cells and different genetic types of CRC.

## The Role of TILs in Anti-Tumor Immunity

It is an increasing variety of investigations that support the importance of tumor immune infiltration, including lymphocytes [T cells, B cells, and natural killer (NK) cells], macrophages, dendritic cells, and neutrophils, revealing a wide patient-patient diversity (19, 20). For a long time in the past, colorectal cancer was regarded as immunogenic and difficult to be treated by immunotherapy. However, advances in the molecular characterization of tumor-associated antigens defined by T cells and methods for detecting antigen-specific T cell responses have changed the scientific community’s view of this issue. Tumors with microsatellite instability, including CRC, accumulate inserts and deletions in DNA repeat sequences. About two-thirds of MSI tumors are sporadic, and one-third are hereditary (Lynch syndrome). The high mutational load and frequent frameshift mutations in MSI tumors lead to the production of many neoantigens recognized by the immune system, which can trigger the lymphocytic infiltrates. Although a portion of TILs is composed of immunosuppressant cells, these cells are specifically recruited and/or directed by the tumor to maintain the immune-privileged microenvironment. In contrast, some TILs reflect attempts by the immune system to counter tumor responses (21, 22).

It is noteworthy that several studies have identified a broad association among TILs, different histological characteristics of cancer, disease-free survival (DFS), cancer-specific survival (CS) and OS (23–26). A meta-analysis of 43 trials describing 21,015 CRC patients showed that high generalized tumor inflammatory infiltrate was associated with good OS (hazard ratio (HR), 0.65; 95% confidence interval (95% CI, 0.54-0.77), CS (HR, 0.58; 95% CI, 0.46-0.73) and DFS (HR, 0.72; 95% CI, 0.60–0.88) (27). Similarly, Rozek et al. found that high TILs (HR = 0.76, 95% CI = 0.64 to 0.89, *p* < 0.001) was favorable prognostic factors for specific and OS in colorectal cancer through a multivariate analysis of 2,369 cases (28). However, the quantity and quality can significantly vary among CRC patients within CRC different MSI statue (29, 30). Next, we reviewed the association between TILs and survival in patients with CRC and the characteristics of major subsets of TILs in the literature with different MSI statue.

## CD8^+^ Cytotoxic T Cells

CD8^+^ cytotoxic T lymphocytes (CTLs), a key component of the adaptive immune system, play an important role in immune defense against intracellular pathogens such as viruses, bacteria and tumors, which were regarded as a major driver of anti-tumor immunity (31, 32). The cytotoxicity process is carried out by several substances produced by CD8^+^ T cells, such as perforin, granzymes, granulysin, Fas ligand, and tumor necrosis factor α (TNF-α) (33, 34). CD8^+^ CTLs mediates tumor rejection by recognizing tumor antigens and directly kill transformed cells. Effector CD8^+^ T cells in the tumor microenvironment generate Interleukin-2 (IL-2), IL-12 and Interferon-γ (IFN-γ), which enhance CD8^+^ CTLs, leading to targeted tumor cell killing (35, 36).

A recent study of most tumor-infiltrating immune cell subtypes revealed that CD8^+^ T cells had the greatest impact on patient survival (37). The role of CD8^+^ CTLs in prognosis was first analyzed in a large cohort of CRC more than 10 years ago (24, 32). Several studies have shown that elevated levels of CTLs in the tumor microenvironment are associated with antitumor effects and improved prognosis in various cancers (30, 38–40). Moreover, tumors from the patient cohorts categorized by a high or low density of immune infiltrate and presence or absence of metastases revealed that adequate immune infiltration with successful initiation and differentiation of CD8^+^ T cells is vital for successful suppression of metastasis development (41).

Microsatellite instability is a good predictor of the prognosis of colorectal cancer, and there is a close relationship between microsatellite instability and the abundance of tumor-infiltrating T-cells (**Table 1**). It is noteworthy that a study of automatic image analysis on 768 colorectal cancers has identified the density of T cell subsets in neoplastic epithelial areas was positively correlated with MSI-H (39). In particular, several immunohistochemistry studies have revealed an especially high infiltration of intraepithelial activated CD8^+^ T cells within microsatellite instability colorectal tumors (42–45). Dolcetti et al. using immunohistochemistry found that there were many cytotoxic infiltrating structures in tumor epithelial cells in MSI-H patients. Moreover, granase B expression showed that these cytotoxic effects were more active in MSI-H tumors (5.3 ± 4.5 vs 0.6 ± 1.3, *p* < 0.001) (46). Similarly, in another study evaluating the number of multiple immune cells in an *in situ* immune response of 490 patients with CRC, the total density of cytotoxic T cells was significantly higher in MSI samples than in MSS samples. Interestingly, due to the importance of accurate intratumoral localization of infiltrating immune cells, the study also measured the density within the tumor glands (intratumoral) or stroma. The group reported that MSI-H and MSS patients showed similar stromal CD8^+^ T cell densities and there was a significant increase in the density of CD8^+^ T cells within the tumor glands in MSI patients, in both the core and invasive margin of tumor (all *p* < 0.05) (47). The same conclusion was also found in the study of Smedt et al., which identified high numbers of intra-epithelial CD8^+^ cells in MSI compared with MSS tumors (48, 49).

**Table 1:** The association of tumor infiltrating lymphocytes with microsatellite stability status in colorectal cancer.

Author	Markers	Sample size (dMMR-MSI-H | pMMR-MSI-L/MSS)	Disease stage	TILs feature
Liu et al. (42)	CD3, CD4, CD8, CD56	167/163	I-IV	dMMR group displayed higher CD8 cells (p < 0.01). CD56^+^ cells CD4^+^ cell than pMMR group (both p < 0.05).
Flahec et al. (43)	CD3, CD4, CD8, CD20, CD68, FOXP3	35/34	I-IV	dMMR tumors have more numerous intraepithelial (CD3^+^, CD8^+^, FOXP3^+^) and stromal (CD8^+^) lymphocytes
Michael-Robinson et al. (44)	CD3, CD8, CD20	32/70	Duke’s stage A-D	TILs were most abundant in MSI-H colorectal cancers in which 23/32 (72%) scored as TILs positive. Only 5/40 (12.5%) MSS tumours and 9/30 (30%) MSI-L cancers were TILs positive (p < 0.0001).
Phillips et al. (45)	CD3, CD4, CD8	26/138	NA	MSI-H tumours showed significantly higher counts for CD3^+^ and CD8^+^ cells, but no differences were found in CD4 counts.
Dolcetti et al. (46)	CD3, CD4, CD8, CD56	18/37	Duke’s A-D	MSI cases carried significantly higher numbers of cytotoxic lymphocytes infiltrating within neoplastic epithelial structures (p < 0.001)
Mlecnik et al. (47)	CD8, CD20, CD68, IL-17, NKp46, CD45RO	186/114	I-IV	A significant increase in cytotoxic T cell, B cell in tumors from MSI patients. MSI tumors had higher densities of Th1. The MSS patients showed a significantly increased Th17 infiltration in the core and invasive margin of tumor (p < 0.05)
Smedt et al. (48)	CD3, CD4, CD8, CD20, CD68	29/27	I-IV	An increased number of tumor-infiltrating cytotoxic T-lymphocytes (CD8^+^) in MSI compared with MSS tumors for both the tumor and peritumoral area. Quantification showed high numbers of intra-epithelial CD3^+^, CD4^+^, CD8^+^, CD20^+^ and CD68^+^ cells in MSI compared with MSS cancers (all p <= 0.01).
Nestarenkaite et al. (49)	CD8, CD20, CD68	39/48	I-IV	The CD8^+^ densities within tumor-stroma interface zone (IZ) and the intratumoral densities were higher in MSI than in MSS tumors, whereas no differences in IZ and intratumoral CD20^+^ cell densities were observed comparing MSI and MSS tumors
Gouvello et al. (50)	IL-17	10/11	I-IV	Higher tumoral expression of Foxp3, IL-17, IL1-beta, IL-6 and TGF-β was associated with the MSS phenotype, and the IL-17 T/TN (colon cancers/autologous normal colon mucosa) ratio was higher in MSS tissues than in MSI-H tissues.
Michel et al. (51)	CD3, CD8, FOXP3	37/33	I-IV and NA	The elevated number of CD8^+^ lymphocytes found in MSI-H colorectal cancers is paralleled by an enhanced infiltration with CD8^-^ FOXP3^+^ cells

Th, T helper; Treg, regulatory T cell; dMMR-MSI-H, mismatch-repair-deficient and microsatellite instability-high; pMMR-MSI-L/MSS, mismatch-repair-proficient and microsatellite-stable or have low levels of microsatellite instability; TILs, tumor infiltrating lymphocytes.

## T Helper Cells

CD4^+^ helper T lymphocytes are mediators of cellular immunity and play a key role in the activation of other immune cells, such as B cells and cytotoxic T cells, modulating immune responses. CD4^+^ helper T cells further differentiate into subsets with broad functions characterized by cytokine secretion and effector function, including T helper 1 (Th1) cells, T helper 2 (Th2) cells, T helper 17 (Th17) cells, follicular helper T (Tfh) cells (reviewed in the later section) and regulatory T (T_reg_) cells.

The main effector function of Th1 cells lie in cell-mediated immunity and inflammation, including the activation of other immune cells such as macrophages, B cells and CD8^+^ CTLs lysis and other effector functions, which play an important role in clearing intracellular infection and assisting in killing tumor cells. Th1 cells and their derived cytokines (e.g., IFN-γ, TNF-α, etc.) are strongly associated with good clinical outcomes in almost all cancer types (21, 25, 41, 52). In contrast to the effects of Th1, analysis of the effect of other CD4^+^ T cell subsets on clinical outcomes has yielded apparent contradictory results, remaining a matter of debate (**Table 2**). The prognostic effects of other T-helper cell populations (Th2, Th17, and T_reg_ cells) are also different across cancer types and stages. Th2 cells are usually associated with aggressive tumors, either by activating B cells or producing the immunosuppressive cytokine IL-10 (55, 56, 86). However, it is not a universal phenomenon. Multiple studies have shown that Th2 cells are associated with a good prognosis in Hodgkin’s lymphoma (53) and breast cancer (54), but not in ovarian (55), gastric (57) and pancreatic cancer (56). There were also conflicting results regarding the role of Th17 cells, which are associated with poor prognosis [e.g., NSCLC (62) and hepatocellular carcinoma (63)] and improved survival [esophageal cancer (58), gastric cancer (59), ovarian cancer (60), cervical cancer (61)]. Th17 cells recruitment have been observed in a variety of malignancies in comparison with normal tissue. On the one hand, the potential of Th17 cells to transdifferentiate into a more immunosuppressive phenotype plays a role in tumor immune evasion. IL-17 (a cytokine produced by Th17 cells) cytokines are associated with increased vascular growth and thus increase tumor growth and metastasis in some models. On the other hand, Th17 cells recruit CTLs and dendritic cells to the tumor site to promote tumor clearance, similar to their ability to convert to a Th1 phenotype that secretes IFN-γ under specific environmental factors (87). The role of Th17 cells in cancer progression appears to be highly dependent on the specific tumor microenvironment. Harnessing this plasticity to control them and improve anti-tumor responses may be a useful strategy for developing cancer immunotherapies. T_reg_ cells can inhibit anti-autoimmune reactions, and there are different subsets (including thymic-derived T_reg_, peripheral T_reg_, etc.). Similarly, the role of regulatory T cells has been a matter of debate for the past decade. Curiel et al. first demonstrated a correlation of intratumoral T_reg_ cells and poor survival in ovarian cancer (70). However, subsequent studies have reported inconsistent results, with T_reg_ cells having no effect on survival of anal squamous cell carcinoma (67), glioma (68), and glioblastoma (69), while showing positive effects on nasopharyngeal cancer (64), head and neck cancer (65), and hematological malignancies (66).

**Table 2:** The association of different types of TILs with tumor prognosis.

The types of TILs	Reference	Prognosis	Tumor types
CD8^+^ cell	(30, 37–40)	Good	Colorectal cancer etc.
Th1 cell	(21, 25, 41, 52)	Good	Colorectal cancer etc.
Th2 cell	(53, 54)	Good	Hodgkin lymphoma; Breast cancer
	(55–57)	Poor	Ovarian cancer; Pancreatic cancer; Gastric cancer
Th17 cell	(58–61)	Good	Esophageal squamous cell carcinoma; Gastric adenocarcinoma; Ovarian cancer; Squamous cervical cancer
	(62, 63)	Poor	Non-small cell lung cancer; Hepatocellular carcinoma
T_reg_ cell	(64–66)	Good	Nasopharyngeal carcinoma; Head and neck cancer; Urinary bladder cancer
	(67–69)	None	Anal squamous cell carcinoma; Glioma; Glioblastomas
	(70)	Poor	Ovarian carcinoma
NK cell	(71–76)	Good	Metastatic prostate cancer; Non-small cell lung cancer; Colorectal cancer; Mantle cell lymphoma
	(77, 78)	Poor	Infiltrating ductal carcinoma of breast; Digestive cancer
B cell	(79–83)	Good	Hepatocellular carcinoma with lymphocytic infiltration; Melanoma; Ovarian cancer; Non-small cell lung cancer; Stage IB cervical squamous cell carcinoma
	(84, 85)	Poor	Ovarian cancer; Breast cancer

A growing number of studies have investigated the characteristics and prognostic potential of T helper cells in CRC adaptive immune response (**Table 1**). A study conducted by Liu and his colleagues showed that the dMMR group displayed much less CD56^+^ cell, CD4^+^ cell and MHC class I expression (all *p* < 0.05) and higher CD8 expression (*p* < 0.01) than the pMMR group. Besides, in the dMMR group, low CD4 and CD56 expression were risk factors for low MHC class I expression in the univariate model (42). However, due to helper cells exhibiting a great diversity in phenotype, identification of the T helper cell subsets in tumors requires evaluation of some specific markers (including, but not limited to, mRNA and key cytokines) in addition to CD4^+^. A study of 52 patients with CRC showed that IL-17 was co-stained with CD4 and CD68 by confocal microscopy analysis, which indicated IL-17 in colorectal cancer was expressed by macrophage and Th17. Compared to T_reg_ cells, other T-helper cell subsets generally do not express distinct surface markers. As a result, several studies have assessed T helper cell abundance through gene expression profiles. In 2013, Bindea et al. performed microarray expression experiments in tumors from 105 CRC patients showed that CD8^+^ and Th1 were associated with a good prognosis (DFS, HR < 1) (88), confirming previous reports from the same group (24). In this report, Th17 cells were also found to negatively influence the patient outcome (DFS, HR > 1, *p* < 0.05). In a large study of 125 frozen colorectal tumor specimens, immune-related genes indicated that patients with high expression of the Th17 cluster had a poor prognosis, whereas patients with high expression of the Th1 cluster had prolonged disease-free survival. In contrast, their results did not support the primary role of Th2 cells in patient outcomes (52). Using single cell RNA-seq, Zhang et al. (89) found that among CD4^+^ T cells, most tumor-infiltrating Treg cells showed clonal exclusivity, while certain Treg cell clones were associated with the development of several T helper cells clones by single T cell transcriptome analysis (89). Notably, two IFNG^+^ Th1-like cell clusters were also found in this study, only CXCL13^+^ BHLHE40^+^ Th1-like cells were preferentially enriched in patients with microsatellite-instable tumors.

T_reg_ cell is characterized by high expression of CD25 and the transcription factor fork head box protein P3 (FOXP3) (64, 90). Using quantitative reverse transcription-PCR (qRT-PCR) quantified for the expression of 15 markers of the immune response, Cui et al. found that higher expression of FOXP3, IL-17, IL1-β, IL-6 and TGF-β were associated with the MSS phenotype (50). Moreover, a large study of 1,420 tumor samples found a significantly higher amount of FOXP3^+^ tumor-infiltrating T_reg_ in pMMR CRC samples (38). This study also observed an association between a high frequency of tumor-infiltrating FOXP3^+^ T_reg_ and improved survival in CRC patients, which is in accordance with the results reported by Gunnarsson et al. and Frey et al. (91, 92). In contrast, several studies have challenged the characterization of T_reg_ cells in CRC. In MSI-H CRC, Michel et al. found a significant increase in intraepithelial infiltration of FOXP3^+^ cells and in the ratio of intraepithelial to stromal infiltration. Similarly, in another study, CD45RO^+^ and FOXP3^+^ cell densities were significantly correlated with MSI-H and the densities of CD8^+^, CD45RO^+^ and FOXP3^+^ cells were significantly associated with patient survival in CRC (43). Given the diversity of T_reg_ populations observed in cancer, it is a great challenge of studying T helper cell subpopulations in the context of immunopathology.

## NK Cells

In recent years, the rapid and potent anti-tumor function of innate immunity, which even occurs at a very early stage of tumor progression, has attracted increasing attention. NK cells, as a subset of innate lymphoid cells, are able to control tumor growth as well as the initial stages of metastatic dissemination (93–95). Unlike other lymphocytes (including B cells, T cells, and natural killer T cells), NK cells do not express antigen-specific receptors such as B cell receptor/T cell receptor or CD3. Instead, NK cells possess cytotoxic abilities similar to CD8^+^ T cells, acting in an antigen-independent manner in the adaptive immunity. In addition to cytotoxic effects, NK cells have been reported to produce a large number of cytokines similar to T cells, including IL-2 (96), IL-7 (97), IL-15 (98), and IFN-γ (99), to modulate adaptive immune responses and participate in other related pathways. Despite many similarities, compared with effector T cells, NK cells are more cytotoxic to tumors, possess lower immunogenicity and respond to target cells more quickly (100, 101). NK cells are highly heterogeneous, characterized by the abundance of surface receptors. According to surface CD56 expression, NK cells can be divided into 2 developmentally related, but functionally distinct, subsets: CD56^bright^ and CD56^dim^. CD56^dim^ NK cells are comprise 80%–95% of peripheral blood NK cells, and are always also CD16^+^, expressing high levels of KIR and LFA-1 and showing cell killing ability (102). However, CD56^bright^ NK cells are traditionally considered ineffective antitumor responders that instead function primarily in immunomodulation, which mainly secrete cytokines such as IFN-γ, TNF-β, and granulote-macrophage colony-stimulating factor (GM-CSF) (103–105). Nevertheless, NK cells in various tissues (106–108), even in the same organ and tissue (109), have diverse features.

The specific role of NK cells, with the complexity of intrinsic signaling pathways, remains controversial in distinct cancer types (**Table 2**). Due to the complex and variable functional status, NK cells were shown to vary survival and therapeutic response in different types of cancer (71, 77, 78, 110–112). In CRC, NK cells have been consistently associated with increased survival in patients (72–76). It is noteworthy that CRC patients with dMMR-MSI-H and pMMR-MSI-L/MSS also seem to display different NK cell features (**Table 1**). The surface markers of NK cells vary greatly and it is difficult to accurately identify NK cell type by one or two simple molecules. However, in many studies, NK cells have been detected using CD56 as a phenotypic marker. Liu et al. assessed for the presence of NK cell infiltration in CRC tissues using the expression of CD56, and found that CD56^+^ cells were reduced in the dMMR group (*p* < 0.05) through immunohistochemical (42). In apparent contrast with these observations, Mlecnik et al. quantified the number of cells by detecting NKp46 and found no significant difference in NK cells between MSI and MSS patients (47). The mechanism of NK cells is complex and variable, and its actual role in the tumor microenvironment remains to be further clarified.

## B Cells, Tfh, and Tertiary Lymphoid Structures

### Tumor-Infiltrating B Lymphocytes

B cells, with a variety of immune functions, are recognized as the main effector cells of the humoral adaptive immune response. However, TIBs can be observed in various solid tumors, but their role in cancer remains controversial (**Table 2**). In HCC (79), melanoma (80), high-grade serous ovarian cancer (81), NSCLC (82) and stage IB cervical squamous cell carcinoma (83), increased B cell count is associated with improved clinical outcomes. However, in epithelial ovarian cancer (84) and breast cancer (85), B cell infiltration is correlated with poor prognosis. At present, studies on the prognostic potential of B cells are limited. It is worth noting that most of the current studies quantify TIBs by CD20. A recently reported systematic review of TIBs into CRC showed that patients whose tumors were highly infiltrated by CD20^+^ B lymphocytes had a significantly improved DFS improvement DFS (HR = 0.45, 95% CI 0.28-0.73, *p* = 0.001). Moreover, the author also found that CD20^+^ B lymphocytes were highly and positively associated with CD8^+^ T lymphocytes (*p* < 0.001) (113). Interestingly, a report demonstrated that an increase in the number of TIBs was associated with improved clinical outcomes for CRC (88). However, another report has given a complex interpretation of the roles of TIBs in CRC (49). It was observed that CD8^+^ and CD20^+^ immunogradient indicators, that reflect cell migration towards the tumor, were associated with improved patient survival, while the infiltrative tumor growth pattern was linked to worse patient outcomes. In addition, this study also found that high numbers of intra-epithelial CD20^+^ cells were observed in MSI tumors compared with MSS tumors and MSS colorectal tumors were characterized by elevated levels of intratumoral CD20^+^.

### Tfh and TLSs

**Figure 1:**
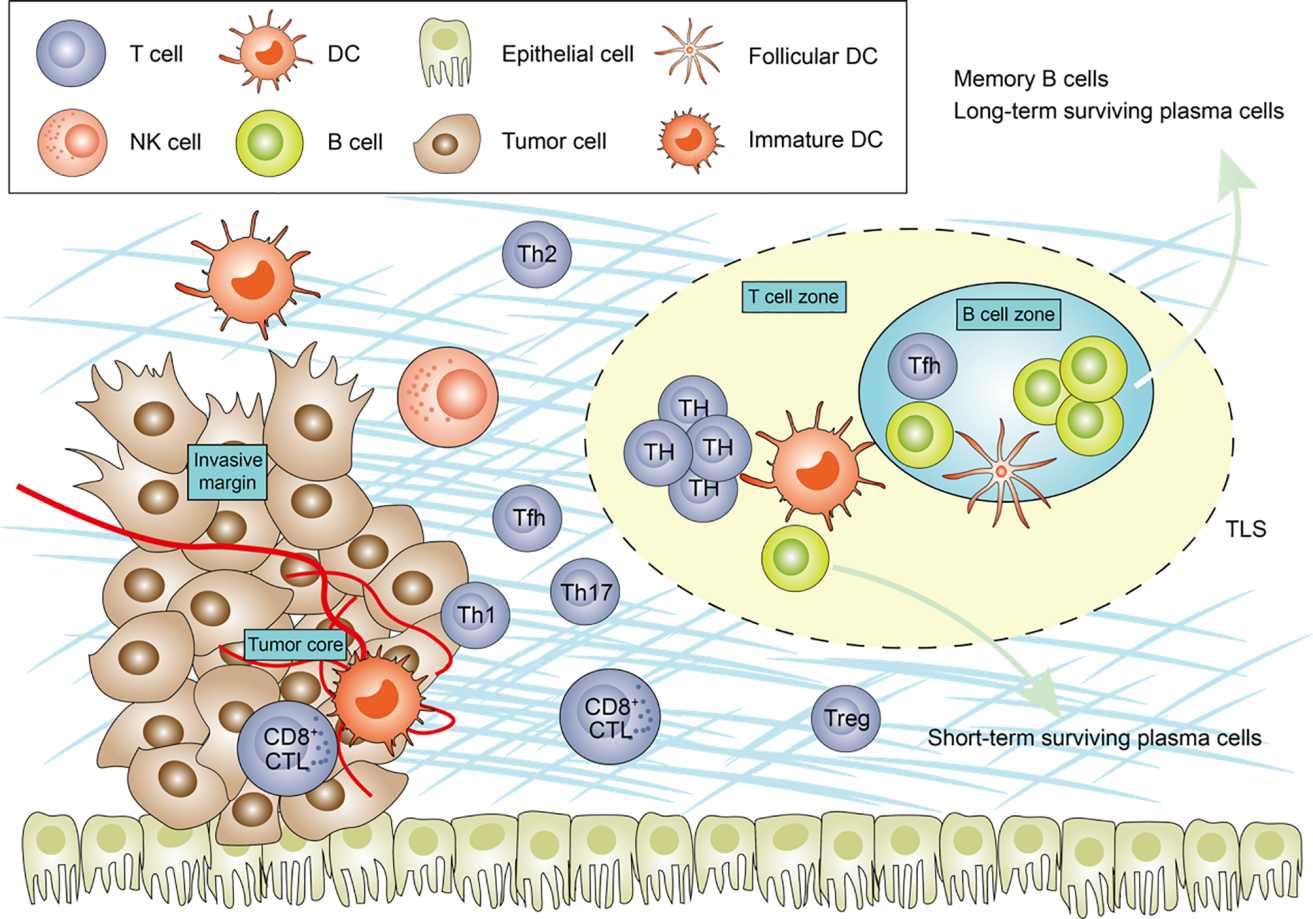
The main tumor-infiltrating lymphocytes and tertiary lymphoid structures components in cancer. The schematic representation shows the features of the immune contexture, including tumor-infiltrating lymphocytes and tertiary lymphoid structures (TLSs). TLSs are usually located in the invasive margin or in the stroma rather than the tumor core. Tfh cells are the most important sources of CXCL13, induced TLSs formation. Th17 cells, B cells have been shown to be able to initiate of TLSs genesis in various pathological contexts. The synergistic effect of CD8^+^ cytotoxic effector T cells and B cells, generated in TLS, enable to direct kill tumour cell. Central memory B cells generated in TLSs protect against metastasis. TH, T helper cell; Treg, regulatory T cell; Tfh, follicular helper T; CTL, cytotoxic T cells; DC, dendritic cell.

Tfh cells, a T helper cells subset, are essential for the maturation and activation of B cells, which are characterized by the expression of CXCR5, an inducible T-cell co-stimulator. B cells, Tfh and related pathways also maintain the structure and function of the tertiary lymphoid structure. The interactions among Tfh cells, B cells and follicular dendritic cells are the basis of the adaptive immune response, which results in B cells differentiating into memory B cells and long-term surviving plasma cells. In addition, B cells can infiltrate into tumors and affect tumor progression through CXCL13 secreted by Tfh and follicular dendritic cells (114, 115). B cells, Tfh and related pathways also maintain the structure and function of the TLS. The current consensus is that the Tfh cell and B cell axis within tumor-associated TLSs contribute to the formation of anti-tumor immune structures (116). TLSs are transient ectopic lymphoid organs that share several structural and functional features with secondary lymphoid organs (117), and consist of B cell follicle and T-cell-rich areas that are sites for the differentiation of T cells and B cells (118) (**Figure 1**). B cell follicle, composed of a core germinal centre containing mostly B cells, but also Tfh cells, follicular DCs and macrophages, surrounded by a ring of naive B cells; and a T-cell-rich area, composed of clusters of T cells and mature DCs (119).

There is increasing evidence that TLS is an effective modulator of immune responses. TLSs are often [NSCLC (120), HER2^+^ breast cancer (121), melanoma (122)], but not always [HCC (123)], associated with favourable clinical outcomes in most types of cancer. In local and metastatic CRCs, TLSs are associated with improved survival and may represent activation of an adaptive immune response to malignant cells (124, 125). In a cohort study involving a consecutive series of 351 patients with stage II and III colorectal cancer, the TLSs density and infiltration of patients with stage II are correlated and coordinated to predict better patient outcomes (126). In addition, the murine model showed an active role of TLSs in the recruitment of lymphocytes to tumor areas. Moreover, certain heterogeneities exist among TLSs from different cancer types, locations and stages. Posch et al. performed a comprehensive molecular, tissue, laboratory, and clinical analysis of 109 patients with stage II/III CRC (127). TLSs were found to be formed in most tumors and were more prevalent in CRC with MSI-H and/or BRAF mutations. In addition, the authors also found that TLSs maturation contained important prognostic information about the risk of disease recurrence. In a recent report examing the cellular composition and association with patients’ prognosis in each TLSs, the authors reported that the densities of T helper cells and macrophages in TLSs were significantly higher in relapsed patients than in not-relapsed patients (*p* = 0.043 and *p* = 0.0076) (128). Multivariate analysis also showed that a high proportion of T helper cells was the most significant independent risk factor for disease recurrence. In contrast, there is little data available regarding Tfh cells supporting anti-tumor responses in CRC. A high expression of Tfh and B cell genes was found strongly associated with a good prognosis in CRC according to Bindea et al. (88). The authors also found that Tfh and intrinsic cell density increased with tumor progression. Obviously, there are clearly interesting complexities to Tfh-associated biology in the context of cancer, and the available data show that much more needs to be learned.

## TILs and Immunotherapy

### T-Cell-Based Immunotherapy

In CRC, T cell infiltration into the tumor has been associated with good outcomes, and prevention of its exhaustion and apoptosis in tumors is the goal of immunotherapy, especially immune checkpoint inhibitors. ICIs target negative costimulation receptors or their ligands of TCR signals, such as CTLA4, PD-1 and PD-L1, to prevent tumor cells attenuate T-cell activation (129).

**Figure 2:**
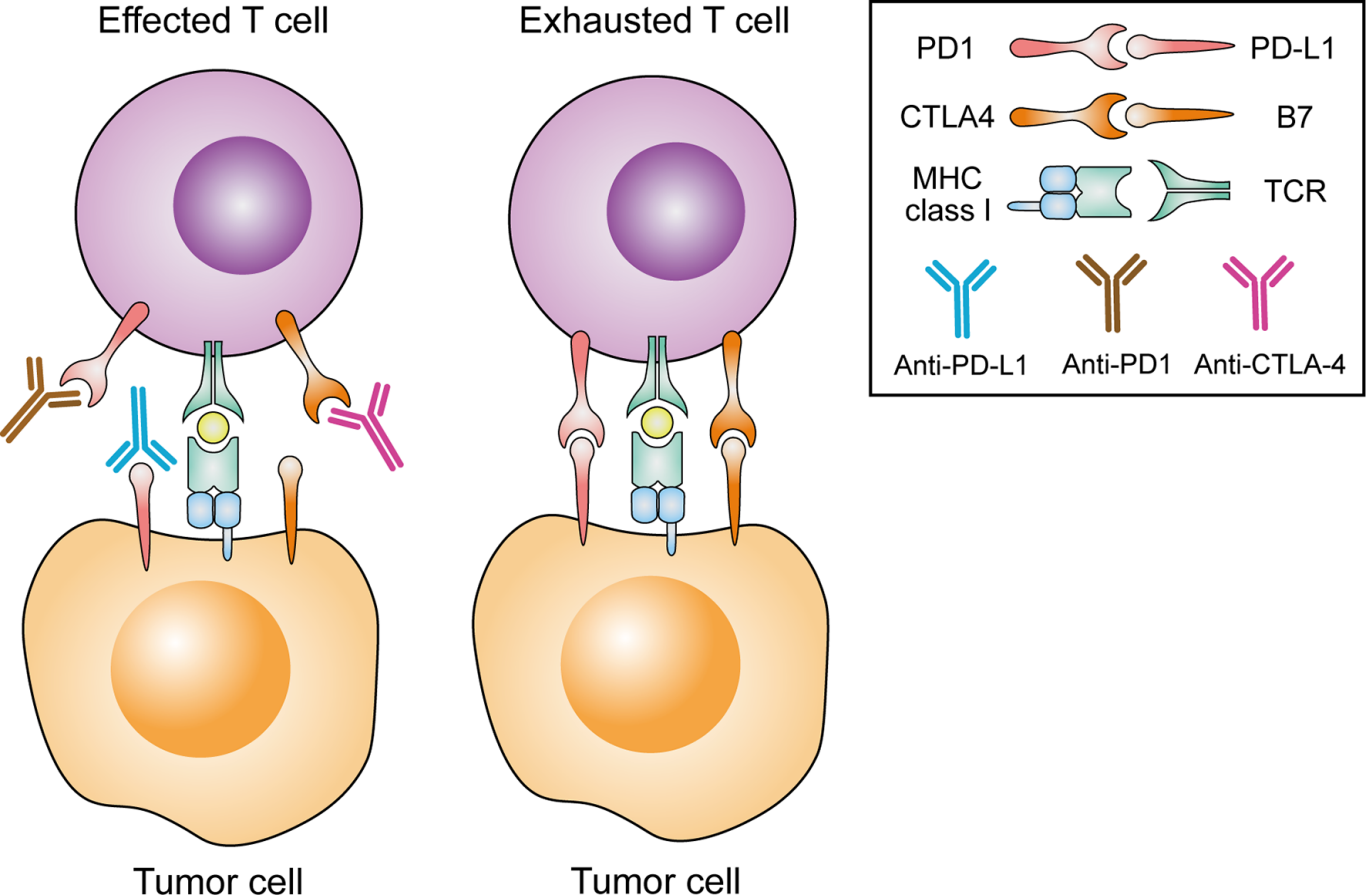
Rationale for the current FDA-approved CRC immune checkpoint inhibitor strategies. TCR, T cell receptor; MHC, major histocompatibility complex; CTLA4, cytotoxic T lymphocyte antigen 4; PD1, programmed cell death 1; PD-L1, programmed cell death 1 ligand 1.

ICIs have shown very limited clinical activity in early studies of CRC treatment (130, 131). In 2015, a phase II study investigated the efficacy of pembrolizumab, a humanized IgG4 antibody directed against surface-expressed PD-1, in three separate cohorts of 41 patients with MSI-H and MSS CRC tumors, and MSI-H tumors from other sites (non-CRC). Results showed that the immune-related objective response rate was 40% (4 had a partial response and 5 had the stable disease) with dMMR-MSI-H patients, whereas there was no objective response in patients with MSS CRC. The median progression-free survival (PFS) and OS were not yet reached in the dMMR-MSI-H cohort but were 2.2 months and 5.0 months, respectively, in the pMMR-MSI-L/MSS cohort (HR for disease progression 0.10 (*p* < 0.001); HR for death 0.22 (*p* = 0.05) (14). Similarly, another study of 53 patients treated with pembrolizumab showed the benefit of immune checkpoint blockade in dMMR-MSI-H tumors. The response rate was 50% (95% CI 31–69%), and the disease control rate was 89% (25/28) in the 28 patients with dMMR-MSI-H tumors. At 24 months, PFS was 61%, and OS was 66%. None of the 18 patients with pMMR-MSI-L/MSS CRC responded and the disease control rate was 16% (4/25) (132). On May 23, 2017, FDA approved pembrolizumab based on the data from 149 patients (84% for colorectal cancer) for the treatment of adult and pediatric patients with unresectable or metastatic, dMMR-MSI-H solid tumors, regardless of tumor site or histology (133) (**Figure 2**). In addition to pembrolizumab, nivolumab, another PD-1 inhibitor, was tested in 74 patients with dMMR-MSI-H metastatic colorectal cancer (134). At a median follow-up duration of 12 months, the objective response rate was 31% (23/74), and in 69% (51/74) patients who had disease control for 12 weeks or longer were observed. In July 2017, FDA expedited approval of nivolumab for the second-line treatment of patients with dMMR-MSI-H CRC.

Compared with patients with dMMR-MSI-H CRC, immunotherapy alone has not shown a clinical benefit in patients with pMMR-MSI-L/MSS CRC. As a result, alternative approaches to immune modulation studies are ongoing. Tumor immune microenvironment, as a critical obstacle to the development of immunotherapy, has been studied with medications that have immunomodulatory properties. Indomethacin 2,3-double oxygenase 1 (IDO1) is an intracellular enzyme that can cause tryptophan depletion, has been reported to play multiple roles in cancer, including inhibiting T and NK cells, producing active Treg and myeloid-derived suppressor cells and promoting tumor angiogenesis (135). Kitsou et al. found that IDO1 was significantly overexpressed in CRC and exhibited anticancer activity (136). In murine intestinal adenomas cell-specific Stat1 deletion models, loss of IDO1^+^ Paneth cells had profound effects on the intratumoral immune cell composition. Moreover, the patient samples and TCGA expression data supported corresponding cells in human colorectal tumors, suggesting IDO1^+^ Paneth cells as a target for immunotherapy (137). Epacadostat, an IDO1 inhibitor, was planned to combinate with pimuzumab and azacytidine in the MSS CRC study. However, this study has been terminated at an early stage. Overall, the molecular of IDO1 inhibitor shows promising anti-tumor potential.

With the knowledge in CRC biology improved, another immunomodulatory strategy, the combination of MEK and PD-L1 inhibition, was developed. In many preclinical studies, inhibition of MEK, a downstream effector of the RAS-MAPK pathway, was found to induce PD-L1 upregulation (138). Preclinical data reported in 2016 showed that 4 of 23 patients with CRC had a partial response, in which three patients had confirmed pMMR-MSI-L (139). In addition, many clinical trials are also studying the combination of MEK inhibitor with anti-PD1 antibody and other chemotherapeutic drugs.

### The Emergence of Natural Killer Cells as a Target in Cancer Immunotherapy

NK cells, as an important natural immune effector, are effector lymphocytes that control several types of tumors and microbial infections. In recent years, research on NK cell-related immunotherapy has been developing vigorously, and a number of NK cell-based therapeutic studies achieved favorable results. Recently, several studies have shown that cytokine supplementation can promote the development and cytotoxicity of NK cells. It has been revealed that direct contact with membrane-bound IL-15 on adjacent stromal cells could induce stronger cytotoxic effects in NK cells in the mice model (140). In a human multicenter phase I study, NKTR-214, a novel IL-2 pathway agonist, showed clinical activity including tumor shrinkage and durable disease stabilization in heavily pretreated patients (141). Moreover, in parallel with CD8^+^ T cells, NK cells can also be suppressed by immune checkpoint molecules. NKG2D, an essential receptor for the activation of NK cells, has been reported to be upregulated by many ligands in tumor cells (142). Andrade et al. designed antibodies targeting the MICA α3 domain and found that these antibodies prevented human cancer cells from loss of cell surface MICA and MICB (NKG2D ligands). In addition, these antibodies inhibited tumor growth in multiple fully immunocompetent mouse models and reduced human melanoma metastases in a humanized mouse model (143). Monalizumab, a clinically used antibody targeting NKG2A, has been developed to promote NK cell function and has shown the potential to enhance the efficacy of anti-PD-1 therapy in MSS metastatic CRC (144). In addition, other antibodies designed specifically for NK cells, such as lirilumab, are also under clinical trials. Overall, a number of studies have elucidated the possible mechanisms of NK cells, paving the way for clinical research into NK cell-based cancer therapies, and lighting up hope for patients currently resistant to T cell-based immunotherapy.

### B Cell-Based Cancer Immunotherapy

In addition to T and NK cells, the development of B cell-based immunotherapy strategies may be effective. By bulk and single-cell RNA sequencing, Helmink et al. observed significantly higher levels of B-cell-related gene (such as *MZB1*, *JCHAIN* and *IGLL5*) expression, increased BCR diversity, and clonal expansion in tumor samples from melanoma patients who responded to ICB treatment than patients who did not (145). Besides, a study of gene expression profiles of 608 different subtypes of soft tissue sarcomas found that B cells are the strongest prognostic factor even in the context of high or low CD8^+^ T cells and cytotoxic contents (146). Considering the relationship between B cells and patient prognosis, enhancing anti-tumor B cell activity or may have an anti-tumor effect. It has been established that TIL B cells support antitumor immunity and promote immunotherapy responses by acting as APCs, producing high-affinity antibodies and secreting antitumor cytokines. Katoh et al. identified sulfated glycosaminoglycans as the main functional B cell antigen and its natural antibodies showed robust growth-suppressive functions against a wide variety of human malignancies (147). Intra-tumoral injection of IL-12 was also shown to activate B cells, leading to good outcomes in HNSCC patients (148). Interestingly, Lu et al. found a subpopulation of B cells, ICOSL^+^ B cells, in breast cancer patients who received neoadjuvant chemotherapy. Moreover, using B-cell specific deletion mice, ICOSL^+^ B cells were found to enhance anti-tumor immunity by enhancing effects that modulate T cell proportions (149). Over the past decade, a population of suppressor B cells, regulatory B (B_reg_) cells, have been shown to play a pivotal role in regulating immune responses involved in cancer. Schioppa et al. identified a population of splenic IL-10 producing B_reg_ cells implicated in the suppression of CD8^+^ T cells, which promoted papilloma development and cancer growth in a mouse model of induced skin carcinogenesis (150). The presence of tumor-induced B_reg_ cells has also been reported. In a variety of tumor types, IL-21 induced Granzyme B-Expressing B_reg_ cells has been found to modulate cellular adaptive immune responses by promoting tumor avoidance mechanisms against anti-tumor immune attack (151). On the other hand, in PDAC mice models with KRAS-mutations, IL35-producing B cells have been reported to play a protumorigenic role, which could be inhibited by CD20 specific monoclonal antibody (152). In advanced CRC, Rituximab, a humanized monoclonal antibody targeting human CD20, apparently reduced the tumor burden (153). All of the above evidence points to the potential of B cell immunotherapy. In the future, new immunotherapy strategies should focus on activating TIL B cells, and how to exploit plasma B cells to promote lymphocyte infiltration and stimulate cytotoxic T cell activation to increase the antitumor immune response.

## Conclusion

In recent years, significant achievements have been witnessed in the field of CRC immunotherapy. CD8^+^ TILs are essential for an effective anti-tumor immune response. Monoclonal antibodies that block immune checkpoints to prevent T cell exhaustion and promote tumor destruction by cytotoxic CD8^+^ T cells, have been shown to be effective in mCRC patients with dMMR-MSI-H. In 2017, pembrolizumab was approved by the FDA for the treatment of all dMMR-MSI-H metastatic solid tumors, becoming the first biomarker-based cancer treatment regimen. However, not all dMMR-MSI-H CRC cases respond to ICIs. Compared with dMMR-MSI-H, pMMR-MSI-L/MSS, which accounts for the majority of CRC with a lower mutation load, also shows an unsatisfactory response to ICIs. In tumors without active immune responses, active induction of immune responses by other immunotherapy methods may be required to achieve tumor control in the vast majority of patients with pMMR-MSI-L/MSS.

Increasing evidence supports the major role of infiltrating immune cells, especially TILs, in tumor control. In addition to CD8^+^ T cells, other TILs also have shown the potential in immunotherapy. CD4^+^ T cells play a key role in enhancing tumor control, both during effector T cell initiation and in the tumor microenvironment. Vaccines designed to induce a CD4 response have shown significant promise in improving clinical outcomes in subgroups of patients with melanoma and breast cancer (154). While several early trials have yielded promising data, further studies are needed to verify its safety and effectiveness. Moreover, a growing number of studies have the potential to improve our understanding of NK and B cells antitumor functions, promising positive research in related fields. With insights gained from trials based on NK and B cells, novel therapeutic strategies will likely help to guide clinicians towards a more personalized treatment for CRC patients.

In conclusion, tumor-infiltrating lymphocytes play a significant role in the tumor immune environment. As the regulatory role of TILs in CRC continues to be elucidated, we anticipate that personalized immunotherapy for CRC patients will be realized, and these advances will further drive the clinical success of immunotherapy.

## Author Contributions

FW and ZB conceptualized the study. FW oversaw the literature review was involved in all aspects of designing and writing the manuscript. ZB and YZ performed the literature review. ZB wrote the manuscript and designed the figures. JX and ZY provided input on the discussion of various sections. All authors contributed to the article and approved the submitted version.

## Funding

This work was supported by the National Natural Science Foundation of China grants 31770827 and 21736002.

## Conflict of Interest

The authors declare that the research was conducted in the absence of any commercial or financial relationships that could be construed as a potential conflict of interest.

## Publisher’s Note

All claims expressed in this article are solely those of the authors and do not necessarily represent those of their affiliated organizations, or those of the publisher, the editors and the reviewers. Any product that may be evaluated in this article, or claim that may be made by its manufacturer, is not guaranteed or endorsed by the publisher.
